# Air pollution and geriatric mental health: perspectives on the COVID-19 pandemic

**DOI:** 10.1017/S1041610220001428

**Published:** 2020-07-01

**Authors:** Rachit Sharma, Mahbub Hossain, Priyanka Pawar, Sonam Sharma

**Affiliations:** 1 GRID Council, Delhi-NCR 201301, India; 2 Department of Health Promotion and Community Health Sciences, School of Public Health, Texas A&M University, TX 77843, USA; 3 Department of Pathology, Kalpana Chawla Government Medical College, Karnal, Haryana 132001, India

In the absence of any vaccine or effective pharmaceutical treatment options for COVID-19, the likelihood of long-term necessity of social restrictions and confinement practices like isolation and quarantine among older adults—the most visible victims of COVID-19—remains high (CBC News, 2020; *The Washington Post*, 2020). Such practices, however, contribute substantially to psychological distress, depression, anxiety, mood disorders, insomnia, stigmatization, reduced self-esteem and self-control, and post-traumatic stress disorder among those confined, and are more pronounced amongst the geriatric population (Holmes *et al.*, 2020; Hossain *et al.*, 2020). This necessitates insight and action into addressing various risk factors that may aggravate such adverse mental health consequences among older adults as they continue to self-isolate amid the COVID-19 pandemic (Holmes *et al.*, 2020).

A growing body of research has linked air pollution, both at ambient and household levels, with an array of mental health disorders such as anxiety, stress, depression, mood disorders, suicidal behavior, cognitive impairment, and dementia among older adults (Braithwaite *et al.*, 2019; Oudin *et al.*, 2018). While ambient air pollution levels have considerably plummeted globally due to reduction in outdoor anthropological activities during the pandemic, the levels of household air pollution (HAP) can be expected to have exponentiated as the majority of the populations are spending greater time indoors and engaging in activities such as cooking, heating, and lighting in greater proportions than the pre-COVID-19 era (Venter *et al.*, 2020). This is especially of concern as more than 3 billion people across the globe continue to rely on highly polluting solid fuels (wood, dung, agricultural residues, coal, charcoal) to meet their household energy demands (World Health Organization, 2018).

Experimental evidence suggests that air pollutants, particularly fine particulate matter (PM_2.5_, particulate matter with an aerodynamic diameter less than 2.5-μm)—the most common and major health deterrent combustion product of solid fuels, can easily translocate to the central nervous system through the olfactory-neural pathway and the cardio-respiratory system to induce neuroinflammation, oxidative stress, microglial activation, cerebrovascular dysfunction, and alterations in the blood–brain barrier (Genc *et al.*, 2012). Among epidemiological investigations, Liu and colleagues, by employing the propensity score matching method, compared the effects of HAP exposure on depression among solid and clean fuel (liquefied gas, natural gas, and electricity) users, using a nationally representative dataset of the older population in China. They found a significantly higher Center for Epidemiologic Studies Depression Scale (CES-D) score and depression among solid fuel users than clean fuel users; with a CES-D score of 0.59 (95% confidence interval [CI]: 0.31, 0.89) and the depression risk odds ratio of 1.26 (95% CI: 1.14, 1.41) among solid fuel users (Liu *et al.*, 2020). In another study, based out of Northern Sweden, Oudin and colleagues assessed the longitudinal association between PM_2.5_ emissions from local residential wood burning and dementia. They found a significant association between the PM_2.5_ exposure from woodsmoke and dementia incidence among older adults, with a hazard ratio of 1.55 (95% CI: 1.00, 2.41) for a 1-μg/m^3^ increase in PM_2.5_ concentration (Oudin *et al.*, 2018).

Therefore, given the plausibility of exacerbation of adverse mental health outcomes due to HAP exposure among older adults, primary prevention should be enabled at the household level itself. The need for maintaining maximum ventilation at homes, particularly whilst undertaking activities involving solid fuel use, should be mass communicated along with improving awareness around HAP and associated mental health risks among older adults and their caregivers. Further, ensuring affordability and robust door-step-delivery mechanisms for clean fuels and systems for older adults would be critical, considering their limited mobility and financial capacities that have been worsened by the pandemic. This can be challenging, especially in developing countries that are highly populated and where social welfare is suboptimal. Strengthening of psychosocial care by optimizing existing mental health services for older adults, alongside preventing their HAP exposures by considering the drivers and barriers of sustained clean fuel use should be prioritized. This would require the engagement of multidisciplinary stakeholders, as it may help prioritize context-specific strategies to meet the mental health and clean fuel needs of the geriatric populace. Lastly, from a research perspective, building psychogeriatric evidence in relation to COVID-19 should incorporate modeling for environmental risk factors, including household air quality and trends in fuel usage. Such research efforts should be contextualized by accounting for personal environmental exposures, socio-economic and ethnocultural differences, and the implications of various psychosocial support interventions extended for older adults during the global crisis.
